# Supplementary material for: DNA methylation in Arabidopsis has a genetic basis and shows evidence of local adaptation
Source: eLife. 2015 May 5;4:e05255. doi: 10.7554/eLife.05255 (PMC4413256; doi:10.7554/eLife.05255)
Supplement: Supplementary file 1. — Multiple sequence alignment of CMT2 sequences from different accessions. DOI: http://dx.doi.org/10.7554/eLife.05255.027 [file elife05255s001.pdf]

Alignment of CMT2 sequences from different Accessions:

_ref = reference (Columbia genotype) _altA = CMT2A-nr, _altB= CMT2B-nr

```
TAIR10          MLSPAKCESEEAQAPLDLHSSSRSEPECLSLVLWCPNPEEAAPSSTRELIKLPDNGEMSL 60
992_ref         MLSPAKCESEEAQAPLDLHSSSRSEPECLSLVLWCPNPEEAAPSSTRELIKLPDNGEMSL 60
6918_ref        MLSPAKCESEEAQAPLDLHSSSRSEPECLSLVLWCPNPEEAAPSSTRELIKLPDNGEMSL 60
6188_altA       MLSPAKCESEEAQAPLDLHSSSRSEPECLSLVLWCPNPEEAAPSSTRELIKLPDNGEMSL 60
1061_altA       MLSPAKCESEEAQAPLDLHSSSRSEPECLSLVLWCPNPEEAAPSSTRELIKLPDNGEMSL 60
6113_altB       MLSPAKCESEEAQAPLDLHSSSRSEPECLSLVLWCPNPEEAAPSSTRELIKLPDNGEMSL 60
6191_altB       MLSPAKCESEEAQAPLDLHSSSRSEPECLSLVLWCPNPEEAAPSSTRELIKLPDNGEMSL 60
                ************************************************************

TAIR10          RRSTTLNCNSPEENGGEGRVSQRKSSRGKSQPLLMLTNGCQLRRSPRFRALHANFDNVCS 120
992_ref         RRSTTLNCNSPEENGGEGRVSQRKSSRGKSQPLLMLTNGCQLRRSPRFRAVHANFDNVCS 120
6918_ref        RRSTTLNCNSPEENGGEGRVSQRKSSRGKSQPLLMLTNGCQLRRSPRFRAVHANFDNVCS 120
6188_altA       RRSTTLNCNSPEENGGEGRVSQRKSSRGKSQPLLMLTNGCQLRRSPRFRALHANFDNVCS 120
1061_altA       RRSTTLNCNSPEENGGEGRVSQRKSSRGKSQPLLMLTNGCQLRRSPRFRAVHANFDNVCS 120
6113_altB       RRSTTLNCNSPEENGGEGRVSQRKSSRGKSQPLLMLTNGCQLRRSPRFRAVHANFDNVCS 120
6191_altB       RRSTTLNCNSPEENGGEGRVSQRKSSRGKSQPLLMLTNGCQLRRSPRFRAVHANFDNVCS 120
                **************************************************:*********

TAIR10          VPVTKGGVSQRKFSRGKSQPLLTLTNGCQLRRSPRFRAVDGNFDSVCSVPVTGKFGSRKR 180
992_ref         VPVTEGGVSQRNSSRGKSQPLLTLTNGCQLRRSPRSRAVDGNFDSVCSVPVTGKFGSRKR 180
6918_ref        VPVTEGGVSQRNSSRGKSQPLLTLTNGCQLRRSPRFRAVDGNFDSVCSVPVTGKFGSRKR 180
6188_altA       VPVTKGGVSQRKFSRGKSQPLLTLTNGCQLRRSPRFRAVDGNFDSVCSVPVTGKFGSRKR 180
1061_altA       VPVTEGGVSQRNSSRGKSQPLLTLTNGCQLRRSPRFRAVDGNFDSVCSVPVTGKFGSRKR 180
6113_altB       VPVTEGGVSQRNSSRGKSQPLLTLTNGCQLRRSPRFKAVDGNFDSVCSVPVTGKFGSRKR 180
6191_altB       VPVTEGGVSQRNSSRGKSQPLLTLTNGCQLRRSPRFKAVDGNFDSVCSVPVTGKFGSRKR 180
                ****:******: ********************** :***********************

TAIR10          KSNSALDKKESSDSEGLTFKDIAVIAKSLEMEIISECQYKNNVAEGRSRLQDPAKRKVDS 240
992_ref         KSNSALDKKESSDSEGLTFKDIAVIAKSLEMEIISECQYKNNVAEGRSKLQDPAKRKVDS 240
6918_ref        KSNSALDKKESSDSEGLTFKDIAVIAKSLEMEIISECQYKNNVAEGRSRLQDPAKRKVDS 240
6188_altA       KSNSALDKKESSDSEGLTFKDIAVIAKSLEMEIISECQYKNNVAEGRSRLQDPAKRKVDS 240
1061_altA       KSNSALDKKESSDSEGLTFKDIAVIAKSLEMEIISECQYKNNVAEGRSRLQDPAKRKVDS 240
6113_altB       KSNSALDKKESSDSEGLTFKDIAVIAKSLEMEIISECQYKNNVAEGRSRLQDPAKRKVDS 240
6191_altB       KSNSALDKKESSDSEGLTFKDIAVIAKSLEMEIISECQYKNNVAEGRSRLQDPAKRKVDS 240
                ************************************************:***********

TAIR10          DTLLYSSINSSKQSLGSNKRMRRSQRFMKGTENEGEENLGKSKGKGMSLASCSFRRSTRL 300
992_ref         DTLLYSSINSSKQSLGSNKRMRRSQRFMKGTENEGEENLGKSKGKGMSLASCSFRRSTRL 300
6918_ref        DTLLSSSINSSKQSLGSNKRMRRSQRFMKGTENEGEENLGKSKGKGMSLASCSFRRSTRL 300
6188_altA       DTLSSSSINSSKQNLGSNKRMRRSQRFMKGTENEGVENLGKSKGKGMSLASCSFRRSTRL 300
1061_altA       DTLSSSSINSSKQNLGSNKRMRRSQRFMKGTENEGVENLGKSKGKGMSLASCSFRRSTRL 300
6113_altB       DTLLYSSINSSKQSLGSNKRMRRSQRFMKGTENEGEENLGKSKGKGMSLASCSFRRSTRL 300
6191_altB       DTLLYSSINSSKQSLGSNKRMRRSQRFMKGTENEGEENLGKSKGKGMSLASCSFRRSTRL 300
                ***  ********.********************** ***********************

TAIR10          SGTVETGNTETLNRRKDCGPALCGAEQVRGTERLVQISKKDHCCEAMKKCEGDGLVSSKQ 360
992_ref         SGTVETGNTETLNRRKDCGPALCGAEQVRGTERLVQISKNDHCCEAMKKCEGDGLVSSKQ 360
6918_ref        SGTVETGNTETLNRRKDCGPALCGAEQVRGTERLVQISKNDHCCEAMKKCEGDGLVSSKQ 360
6188_altA       SGTVETGNTETLNRRKDCGPALCGAEQVRGTERLVQISKNDHCCEAMKKCEGDGLVSSKQ 360
1061_altA       SGTVETGNTETLNRRKDCGPALCGAEQVRGTERLVQISKNDHCCEAMKKCEGDGLVSSKQ 360
6113_altB       SGTVETGNTETLNRRKDCGPALCGAEQVRGTERLVQISKNDHCCEAMKKCEGDGLVSSKQ 360
6191_altB       SGTVETGNTETLNRRKDCGPALCGAEQVRGTERLVQISKNDHCCEAMKKCEGDGLVSSKQ 360
                ***************************************:********************

TAIR10          ELLVFPSGCIKKTVNGCRDRTLGKPRSSGLNTDDIHTSSLKISKNDTSNGLTMTTALVEQ 420
992_ref         ELLVFPSGCIKKTVNGCRDRTLGKPRSSGLNTDDIHTSSLKISKNDTSNGLTMTTALVEQ 420
6918_ref        ELLVFPSGCIKKTVNVCRDRTLGKPRSSGLNTDDIHTSSLKISKNDTSNGLTMTTALVEQ 420
6188_altA       ELLVFPSGCIKKTVNVCRDRTLGKPRSSGLNTDDIHTSSLKISKNGTSNGLTMTTALVEQ 420
1061_altA       ELLVFPSGCIKKTVNVCRDRTLGKPRSSGLNTDDIHTSSLKISKNGTSNGLTMTTALVEQ 420
6113_altB       ELLVFPSGCIKKTVNGCRDRTLGKPRSSGLNTDDIHTSSLKISKNDTSNGLTMTTALVEQ 420
6191_altB       ELLVFPSGCIKKTVNGCRDRTLGKPRSSGLNTDDIHTSSLKISKNDTSNGLTMTTALVEQ 420
                *************** *****************************.**************

TAIR10          DAMESLLQGKTSACGAADKGKTREMHVNSTVIYLSDSDEPSSIEYLNGDNLTQVESGSAL 480
992_ref         DAMESLLQGKTSACGAADKGKTREMHVNSTVIYLSDSDEPSSIEYLNGDNLTQVESGSAL 480
6918_ref        DAMESLLQGKTSACGAADKGKTREMHVNSTVIYLSDSDEPSSIEYLNGDNLTQVESGSAL 480
6188_altA       DAMESLLQGKTSACGAADKGKTREMHVNSTVIYLSDSDEPSSIEYLNGDNLTQVKSGSAL 480
1061_altA       DAMESLLQGKTSACGAADKGKTREMHVNSTVIYLSDSDEPSSIEYLNGDNLTQVKSGSAL 480
6113_altB       DAMESLLQGKTSACGAADKGKTREMHVNSTVIYLSDSDEPSSIEYLNGDNLTQVESGSAL 480
6191_altB       DAMESLLQGKTSACGAADKGKTREMHVNSTVIYLSDSDEPSSIEYLNGDNLTQVESGSAL 480
                ******************************************************:*****
```

```
TAIR10       SSGGNEGIVSLDLNNPTKSTKRKGKRVTRTAVQEQNKRSICFFIGEPLSCEEAQERWRWR 540
992_ref      SSGGNEGIVSLDLNNPTKSTNRKGKRVTRTAVQEQNKRSICFFIGEPLSCEEAQERWRWR 540
6918_ref     SSGGNEGIVSLDLNNPTKSTKRKGKRVTRTAVQEQNKRSICFFIGEPLSCEEAQERWRWR 540
6188_altA    SSGGNEGIVSLDLNNPTKSTKRKGKRVTRTAVQEQNKRSICFFIGEPLSCEEAQERWRWR 540
1061_altA    SSGGNEGIVSLDLNNPTKSTKRKGKRVTRTAVQEQNKRSICFFIGEPLSCEEAQERWRWR 540
6113_altB    SSGGNEGIVSLDLNNPTKSTKRKGKRVTRTAVQEQNKRSICFFIGEPLSCEEAQERWRWR 540
6191_altB    SSGGNEGIVSLDLNNPTKSTKRKGKRVTRTAVQEQNKRSICFFIGEPLSCEEAQERWRWR 540
             ********************:***************************************

TAIR10       YELKERKSKSRGQQSEDDEDKIVANVECHYSQAKVDGHTFSLGDFAYIKGEEEETHVGQI 600
992_ref      YELKERKSKSRGQQSEDDEDKIVANVECHYSQAKVDGHTFSLGDFAYIKGEEEETHVGQI 600
6918_ref     YELKERKSKSRGQQSEDDEDKIVANVECHYSQAKVDGHTFSLGDFAYIKGEEEETHVGQI 600
6188_altA    YELKERKSKSRGQQSEDDEDKIVANVECHYSQAKVDGHTFSLGDFAYIKGEEEETHVGQI 600
1061_altA    YELKERKSKSRGQQSEDDEDKIVANVECHYSQAKVDGHTFSLGDFAYIKGEEEETHVGQI 600
6113_altB    YELKERKSKSRGQQSEDDEDKIVANVECHYSQAKVDGHTFSLGDFACIKGEEEETHVGQI 600
6191_altB    YELKERKSKSRGQQSEDDEDKIVANVECHYSQAKVDGHTFSLGDFACIKGEEEETHVGQI 600
             ********************************************** *************
                                  BAH Domain              Y586C
TAIR10       VEFFKTTDGESYFRVQWFYRATDTIMERQATNHDKRRLFYSTVMNDNPVDCLISKVTVLQ 660
992_ref      VEFFKTTDGESYFRVQWFYRATDTIMERQATNHDKRRLFYSTVMNDNPVDCLISKVTVLQ 660
6918_ref     VEFFKTTDGESYFRVQWFYRATDTIMERQATNHDKRRLFYSTVMNDNPVDCLISKVTVLQ 660
6188_altA    VEFFKTTDGESYFRVQWFYRATDTIMERQATNHDKRRLFYSTVMNDNPVDCLISKVTVLQ 660
1061_altA    VEFFKTTDGESYFRVQWFYRATDTIMERQATNHDKRRLFYSTVMNDNPVDCLISKVTVLQ 660
6113_altB    VEFFKTTDGESYFRVQWFYRATDTIMERQATNHDKRRLFYSTVMNDNPVDCLISKVTVLQ 660
6191_altB    VEFFKTTDGESYFRVQWFYRATDTIMERQATNHDKRRLFYSTVMNDNPVDCLISKVTVLQ 660
             ************************************************************

TAIR10       VSPRVGLKPNSIKSDYYFDMEYCVEYSTFQTLRNPKTSENKLECCADVVPTESTESILKK 720
992_ref      VSPRAGLKPNSIKSDYYFDMEYCVEYSTFQTLRNPKTSENKLECWADVVPTESTESILKK 720
6918_ref     VSPRAGLKPNSIKSDYYFDMEYCVEYSTFQTLRNPKTSENKLECCADVVPTKSTESILKK 720
6188_altA    VSPRAGLKPNSIKSDYYFDMEYCVEYSTFQTLRNPKTSENKLECCADVVPTESTESILKK 720
1061_altA    VSPRAGLKPNSIKSDYYFDMEYCVEYSTFQTLRNPKTSENKLECCADVVPTESTESILKK 720
6113_altB    VSPRAGLKPNSIKSDYYFDMEYCVEYSTFQTLRNPKTSENKLECCADVVPTKSTESILKK 720
6191_altB    VSPRAGLKPNSIKSDYYFDMEYCVEYSTFQTLRNPKTSENKLECCADVVPTKSTESILKK 720
             ****.*************************************** ******:********

TAIR10       KSFSGELPVLDLYSGCGGMSTGLSLGAKISGVDVVTKWAVDQNTAACKSLKLNHPNTQVR 780
992_ref      KSFSGELPVLDLYSGCGGMSTGLSLGAKISGVDVVTKWAVDQNTAACKSLKLNHPNTQVR 780
6918_ref     KSFSGELPVLDLYSGCGGMSTGLSLGAKISGVDVVTKWAVDQNTAACKSLKLNHPNTQVR 780
6188_altA    KSFSGELPVLDLYSGCGGMSTGLSLGAKISGVDVVTKWAVDQNKAACKSLKLNHPNTQVR 780
1061_altA    KSFSGELPVLDLYSGCGGMSTGLSLGAKISGVDVVTKWAVDQNKAACKSLKLNHPNTQVR 780
6113_altB    KSFSGELPVLDLYSGCGGMSTGLSLGAKISGVDVVTKWAVDQNTAACKSLKLNHPNTQVR 780
6191_altB    KSFSGELPVLDLYSGCGGMSTGLSLGAKISGVDVVTKWAVDQNTAACKSLKLNHPNTQVR 780
             *******************************************.****************

TAIR10       NDAAGDFLQLLKEWDKLCKRYVFNNDQRTDTLRSVNSTKETSGSSSSSDDDSDSEEYEVE 840
992_ref      NDAAGDFLQLLKEWDKLCKRYVFNNDQRTDTLRSVNSTKETSESSSSSDDDSDSEEYEVE 840
6918_ref     NDAAGDFLQLLKEWDKLCKRYVFNNDQRTDTLRSVNSTKETSESSSSSDDDSDSEEYEVE 840
6188_altA    NDAAGDFLQLLKEWDKLCKRYVFNNDQRTDTLRSVNSTKETSESSSSSDDDSDSEEYEVE 840
1061_altA    NDAAGDFLQLLKEWDKLCKRYVFNNDQRTDTLRSVNSTKETSESSSSSDDDSDSEEYEVE 840
6113_altB    NDSAGDFLQLLKEWDKLCKRYVFNNDQRTDTLRSVNSTKETSESSSSSDDDSDSEEYEVE 840
6191_altB    NDSAGDFLQLLKEWDKLCKRYVFNNDQRTDTLRSVNSTKETSESSSSSDDDSDSEEYEVE 840
             **:*************************************** *****************
                                 CHROMO Domain
TAIR10       KLVDICFGDHDKTGKNGLKFKVHWKGYRSDEDTWELAEELSNCQDAIREFVTSGFKSKIL 900
992_ref      KLVDICFGDPDKTGKNGLKFKVHWKGYRSDEDTWELAEELSNCQDAIREFVTSGFKSKIL 900
6918_ref     KLVDICFGDPDKTGKNGLKFKVHWKGYRSDEDTWELAEELSNCQDAIREFVTSGFKSKIL 900
6188_altA    KLVDICFGDPDKTGKNGLKFKVHWKGYRSDEDTWELAEELSNCQDAIREFVTSGFKSKIL 900
1061_altA    KLVDICFGDPDKTGKNGLKFKVHWKGYRSDEDTWELAEELSNCQDAIREFVTSGFKSKIL 900
6113_altB    KLVDICFGDPDKTGKNGLKFKVHWKGYRSDEDTWELAEELSNCQDAIREFVTSGFKSKIL 900
6191_altB    KLVDICFGDPDKTGKNGLKFKVHWKGYRSDEDTWELAEELSNCQDAIREFVTSGFKSKIL 900
             ********* **************************************************
                      C5 Methyltransferase Domain
TAIR10       PLPGRVGVICGGPPCQGISGYNRHRNVDSPLNDERNQQIIVFMDIVEYLKPSYVLMENVV 960
992_ref      PLPGRVGVICGGPPCQGISGYNRHRNVDSPLNDERNQQIIVFMDIVEYLKPSYVLMENVV 960
6918_ref     PLPGRVGVICGGPPCQGISGYNRHRNVDSPLNDERNQQIIVFMDIVEYLKPSYVLMENVV 960
6188_altA    PLPGRVGVICGGPPCQGISGYNRHRNVDSPLNDERNQQIIVFMDIVEYLKPSYVLMENVV 960
1061_altA    PLPGRVGVICGGPPCQGISGYNRHRNVDSPLNDERNQQIIVFMDIVEYLKPSYVLMENVV 960
6113_altB    PLPGRVGVICGGPPCQGISGYNRHRNVDSPLNDEKNQQIIVFMDIVEYLKPSYVLMENVV 960
6191_altB    PLPGRVGVICGGPPCQGISGYNRHRNVDSPLNDEKNQQIIVFMDIVEYLKPSYVLMENVV 960
             **********************************:*************************
                                               R934K
TAIR10       DILRMDKGSLGRYALSRLVNMRYQARLGIMTAGCYGLSQFRSRVFMWGAVPNKNLPPFPL 1020
992_ref      DILRMDKGSLGRYALSRLVNMRYQARLGIMTAGCYGLSQFRSRVFMWGAVPNKNLPPFPL 1020
6918_ref     DILRMDKGSLGRYALSRLVNMRYQARLGIMTAGCYGLSQFRSRVFMWGAVPNKNLPPFPL 1020
6188_altA    DILRMDKGSLGRYALSRLVNMRYQARLGIMTAGCYGLSQFRSRVFMWGAVPNKNLPPFPL 1020
1061_altA    DILRMDKGSLGRYALSRLVNMRYQARLGIMTAGCYGLSQFRSRVFMWGAVPNKNLPPFPL 1020
6113_altB    DILRMDKGSLGRYALSRLVNMRYQARLGIMTAGCYGLSQFRSRVFMWGAVPNKNLPPFPL 1020
6191_altB    DILRMDKGSLGRYALSRLVNMRYQARLGIMTAGCYGLSQFRSRVFMWGAVPNKNLPPFPL 1020
             ************************************************************
```

```
TAIR10          PTHDVIVRYGLPLEFERNVVAYAEGQPRKLEKALVLKDAISDLPHVSNDEDREKLPYESL 1080
992_ref         PTHDVIVRYGLPLEFERNVVAYAEGQPRKLEKALVLKDAISDLPHVSNDEDREKLPYESL 1080
6918_ref        PTHDVIVRYGLPLEFERNVVAYAEGQPRKLEKALVLKDAISDLPHVSNDEDREKLPYESL 1080
6188_altA       PTHDVIVRYGLPLEFERNVVAYAEGQPRKLEKALVLKDAISDLPHVSNDEDREKLPYESL 1080
1061_altA       PTHDVIVRYGLPLEFERNVVAYAEGQPRKLEKALVLKDAISDLPHVSNDEDREKLPYESL 1080
6113_altB       PTHDVIVRYGLPLEFERNVVAYAEGQPRKLEKALVLKDAISDLPHVSNEEDREKLPYESL 1080
6191_altB       PTHDVIVRYGLPLEFERNVVAYAEGQPRKLEKALVLKDAISDLPHVSNEEDREKLPYESL 1080
                ************************************************:***********
                                                             D1068E
TAIR10          PKTDFQRYIRSTKRDLTGSAIDNCNKRTMLLHDHRPFHINEDDYARVCQIPKRKGANFRD 1140
992_ref         PKTDFQRYIRSTKRDLTGSAIDNCNKRTMLLHDHRPFHINEDDYARVCQIPKRKGANFRD 1140
6918_ref        PKTDFQRYIRSTKRDLTGSAIDNCNKRTMLLHDHRPFHINEDDYARVCQIPKRKGANFRD 1140
6188_altA       PKTDFQRYIRSTKRDLTGSAIDNCNKRTMLLHDHRPFHINEDDYARVRQIPKRKGANFRD 1140
1061_altA       PKTDFQRYIRSTKRDLTGSAIDNCNKRTMLLHDHRPFHINEDDYARVRQIPKRKGANFRD 1140
6113_altB       PKTDFQRYIRSTKRDLTGSAIDNCNKRTMLLHDHRPFHINEDDYARVCQIPKRKGANFRD 1140
6191_altB       PKTDFQRYIRSTKRDLTGSAIDNCNKRTMLLHDHRPFHINEDDYARVCQIPKRKGANFRD 1140
                *********************************************** ************
                                                             C1127R
TAIR10          LPGLIVRNNTVCRDPSMEPVILPSGKPLVPGYVFTFQQGKSKRPFARLWWDETVPTVLTV 1200
992_ref         LPGIIVRNNTVCRDPSMEPVILPSGKPLVPGYVFTFQQGKSKRPFARLWWDETVPTVLTV 1200
6918_ref        LPGLIVRNNTVCRDPSMEPVILPSGKPLVPGYVFTFQQGKSKRPFARLWWDETVPTVLTV 1200
6188_altA       LPGLIVRNNTVCRDPSMEPVILPSGKPLVPGYVFTFQQGKSKRPFARLWWDETVPTVLTV 1200
1061_altA       LPGLIVRNNTVCRDPSMEPVILPSGKPLVPGYVFTFQQGKSKRPFARLWWDETVPTVLTV 1200
6113_altB       LPGLIVRNNTVCRDPSMEPVILPSGKPLVPGYVFTFQQGKSKRPFARLWWDETVPTVLTV 1200
6191_altB       LPGLIVRNNTVCRDPSMEPVILPSGKPLVPGYVFTFQQGKSKRPFARLWWDETVPTVLTV 1200
                ***:********************************************************

TAIR10          PTCHSQALLHPEQDRVLTIRESARLQGFPDYFQFCGTIKERYCQIGNAVAVSVSRALGYS 1260
992_ref         PTCHSQALLHPEQDRVLTIRESARLQGFPDYFQFCGTIKERYCQIGNAVAVSVSRALGYS 1260
6918_ref        PTCHSQALLHPEQDRVLTIRESARLQGFPDYFQFCGTIKERYCQIGNAVAVSVSRALGYS 1260
6188_altA       PTCHSQALLHPEQDRVLTIRESARLQGFPDYFQFCGTIKERYCQIGNAVAVSVSRALGYS 1260
1061_altA       PTCHSQALLHPEQDRVLTIRESARLQGFPDYFQFCGTIKERYCQIGNAVAVSVSRALGYS 1260
6113_altB       PTCHSQALLHPEQDRVLTIRESARLQGFPDYFQFCGTIKERYCQIGNAVAVSVSRALGYS 1260
6191_altB       PTCHSQALLHPEQDRVLTIRESARLQGFPDYFQFCGTIKERYCQIGNAVAVSVSRALGYS 1260
                ************************************************************

TAIR10          LGMAFRGLARDEHLIKLPQNFSHSTYPQLQETIPH 1295
992_ref         LGMAFRGLARDEHLIKLPQNFSHSTYPQLQETIPH 1295
6918_ref        LGMAFRGLARDEHLIKLPQNFSHSTYPQLQETIPH 1295
6188_altA       LGMAFRGLARDEHLIKLPQNFSHSTYPQLQETIPH 1295
1061_altA       LGMAFRGLARDEHLIKLPQNFSHSTYPQLQETIPH 1295
6113_altB       LGMAFRGLARHEHLIKLPQNFSHSTYPQLQEAIPH 1295
6191_altB       LGMAFRGLARHEHLIKLPQNFSHSTYPQLQEAIPH 1295
                **********.********************:***
```
